# A deep learning radiomics model based on CT images for predicting the biological activity of hepatic cystic echinococcosis

**DOI:** 10.3389/fphys.2024.1426468

**Published:** 2024-08-08

**Authors:** Mayidili Nijiati, Mireayi Tuerdi, Maihemitijiang Damola, Yasen Yimit, Jing Yang, Adilijiang Abulaiti, Aibibulajiang Mutailifu, Diliaremu Aihait, Yunling Wang, Xiaoguang Zou

**Affiliations:** ^1^ Department of Radiology, The Fourth Affiliated Hospital of Xinjiang Medical University Ürümqi, Xinjiang, China; ^2^ Department Xinjiang Key Laboratory of Artificial Intelligence Assisted Imaging Diagnosis, Kashi, China; ^3^ Department of Infectious Diseases, The First People’s Hospital of Kashi Prefecture, Kashi, China; ^4^ Department of Radiology, The First People’s Hospital of Kashi Prefecture, Kashi, China; ^5^ Huiying Medical Imaging Technology, The Fourth Affiliated Hospital of Xinjiang Medical University, Beijing, China; ^6^ Department of Imaging Center, The First Affiliated Hospital of Xinjiang Medical University, Ürümqi, China; ^7^ Clinical Medical Research Center, The First People’s Hospital of Kashi Prefecture, Kashi, China

**Keywords:** hepatic cystic echinococcosis, biological activity grading, radiomics, deep learning, 3D-ResNet

## Abstract

**Introduction:** Hepatic cystic echinococcosis (HCE) is a widely seen parasitic infection. Biological activity is crucial for treatment planning. This work aims to explore the potential applications of a deep learning radiomics (DLR) model, based on CT images, in predicting the biological activity grading of hepatic cystic echinococcosis.

**Methods:** A retrospective analysis of 160 patients with hepatic echinococcosis was performed (127 and 33 in training and validation sets). Volume of interests (VOIs) were drawn, and radiomics features and deep neural network features were extracted. Feature selection was performed on the training set, and radiomics score (Rad Score) and deep learning score (Deep Score) were calculated. Seven diagnostics models (based on logistic regression algorithm) for the biological activity grading were constructed using the selected radiomics features and two deep model features respectively. All models were evaluated using the receiver operating characteristic curve, and the area under the curve (AUC) was calculated. A nomogram was constructed using the combined model, and its calibration, discriminatory ability, and clinical utility were assessed.

**Results:** 12, 6 and 10 optimal radiomics features, deep learning features were selected from two deep learning network (DLN) features, respectively. For biological activity grading of hepatic cystic echinococcosis, the combined model demonstrated strong diagnostic performance, with an AUC value of 0.888 (95% CI: 0.837–0.936) in the training set and 0.876 (0.761–0.964) in the validation set. The clinical decision analysis curve indicated promising results, while the calibration curve revealed that the nomogram’s prediction result was highly compatible with the actual result.

**Conclusion:** The DLR model can be used for predicting the biological activity grading of hepatic echinococcosis.

## 1 Introduction

Echinococcosis is a widespread zoonosis that remains a significant public health concern. The two most prevalent forms of this illness, caused by Echinococcu granulosus (E.granulosus) and Echinococcus multilocularis (E.multilocularis), respectively, are alveolar echinococcosis (AE) and cystic echinococcosis (CE) (Deplazes et al., 2017; Faucher et al., 2017; Wen et al., 2019; Yimit et al., 2023). With space-occupying growth for CE and “cancer-like” infiltration growth for AE lesion, the liver is the intermediate host’s most targeted organ. If ignored or poorly managed, AE mortality might approach 90% within 15 years after the first diagnosis. Although mortality for CE is lower than AE, it might significantly increase in individuals who do not receive effective therapy (Kantarci et al., 2012; McManus et al., 2012; Meinel et al., 2018). Of the estimated two to three million cases of echinococcosis globally, most are cystic (Craig et al., 2007).

Clinical judgment heavily relies on the viability assessment of parasite lesions. The World Health Organization Informal Working Group on Echinococcosis (WHO-IWGE) reports that the current method for determining lesion activity ultrasonography and positron emission tomography (PET) scan for parasitic lesion surgical pathology is the gold standard. However, the majority of patients in places with little resources do not have access to or cannot afford this treatment particularly PET scans (Bold et al., 2018). So, it seems sense to look for less expensive, more accessible substitute method.

CT, as a regular imaging modality plays a pivotal role in the comprehensive management of hepatic cystic echinococcosis by facilitating accurate diagnosis, staging, treatment planning, monitoring response to therapy, guiding interventional procedures, and assessing postoperative outcomes. Its detailed imaging capabilities contribute significantly to the effective management and improved prognosis of patients with this parasitic infection (Rinaldi et al., 2014; Graeter et al., 2016).

Radiomics is an emerging technology that combines a large amount of medical image data with human physiological and pathological information, and analyzes medical images to achieve quantitative identification and prediction of diseases (Lambin et al., 2012). Deep learning, a machine learning technique rooted in artificial neural networks, has the capability to autonomously learn and extract significant features from vast amounts of data, leading to its extensive applications. In the field of medical imaging, radiomics and deep learning technologies have been widely employed for the diagnosis, treatment, and prognosis evaluation of various diseases including lung cancer, breast cancer, and stroke (Bera et al., 2022; Chen et al., 2022). In previous studies, radiomics and deep learning were more often used as an independent technology for clinical model construction. In fact, radiomics can be combined with deep learning to construct a DLR combined model. The DLR model can use both human prior manual features (radiomics features) and human unknown deep features extracted by deep neural networks, which makes the feature set more abundant. Recent research has shown promising outcomes using the DLR model. For instance, an international multicenter study utilized the DLR model o estimate the count of lymph node metastases, achieving an AUC value of 0.822 (0.756–0.887) in the validation set (Dong et al., 2020).

In another retrospective study (Jiang et al., 2021), the authors developed a DLR model for predicting pathological complete response after neoadjuvant chemotherapy based on ultrasound images of breast cancer. The model accurately determined pCR status, yielding an area under the receiver operator characteristic curve of 0.94 (95% confidence interval, 0.91–0.97) in the validation set, and was well calibrated. However, there are no reports on DLR method in the grading diagnosis model of echinococcosis biological activity. In this research, our aim is to construct a prediction model for the biological activity grading of hepatic cystic echinococcosis.

## 2 Materials and methods

The study conducted at The First People’s Hospital of Kashi Prefecture and received ethical approval from the institutional review board of The First People’s Hospital of Kashi Prefecture. Since the study was retrospective, written informed consent was deemed unnecessary.

### 2.1 Patients

**FIGURE 1:**
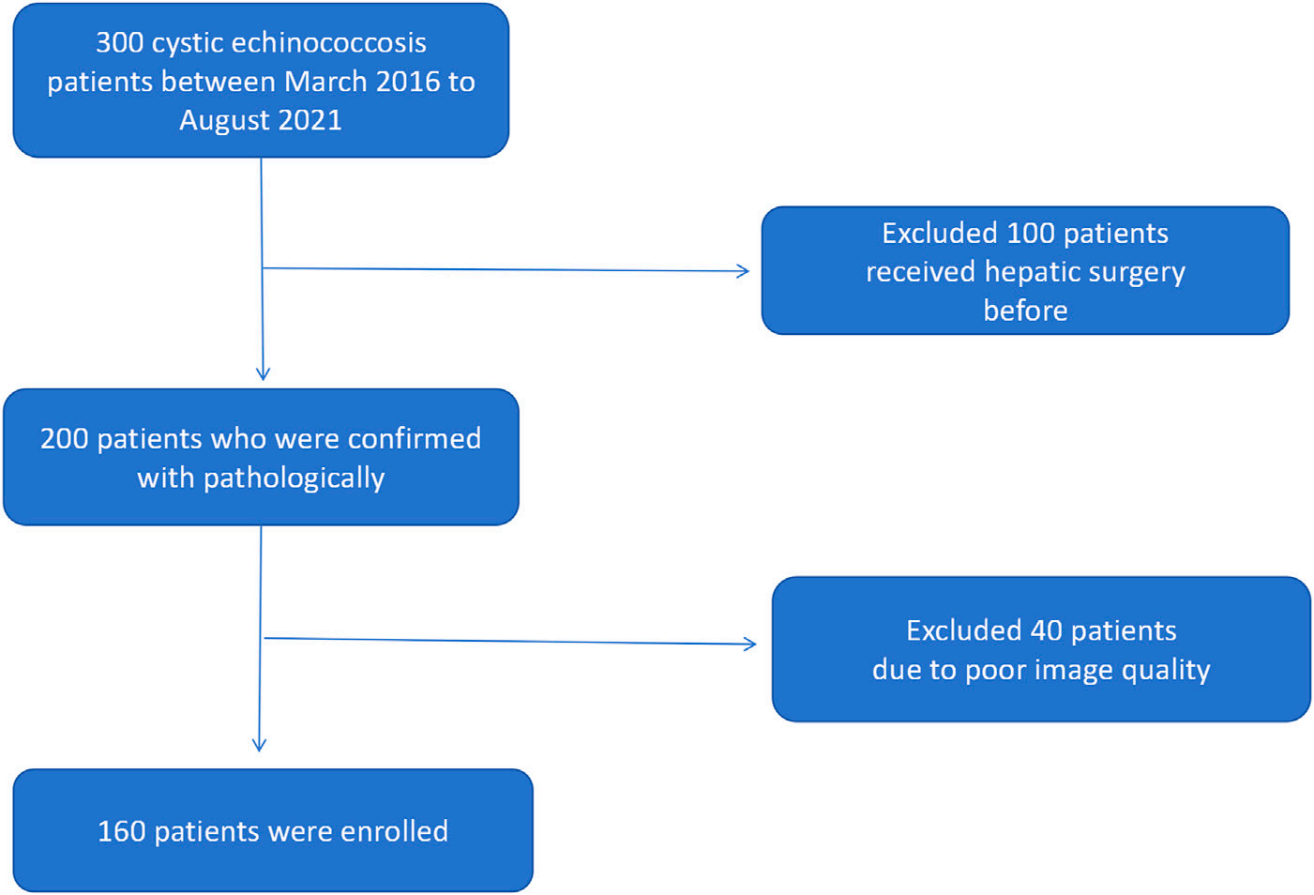
Demonstrates inclusion and exclusion workflow.

We have collected 160 patients who received surgery and confirmed with hepatic cystic echinococcosis pathologically in The First People’s Hospital of Kashi Prefecture between March 2016 to August 2021. The following were inclusion criteria: 1) confirmed with hepatic cystic echinococcosis active or non-active pathologically and clinically 2) patients who did not receive any clinical intervention (chemotherapy or surgery) 3) CT scan was performed; imaging and clinical data were available. The following were exclusion criteria: 1) patients did not underwent surgical treatment, no pathologic confirmation 2) clinical and imaging data were not available 3) has hepatic surgery or anti-parasitic treatment before Figure 1. Demonstrates inclusion and exclusion workflow.

### 2.2 Image acquisition

The Siemens CT scanner was used on all patients. The CT settings were: 5 mm/s bed entry speed, 120 kV voltage, with180–240 mA current, thin slice thickness of 2–3 mm, slice distance of 2–3 mm, and typical slice thickness of 10 mm.

### 2.3 Segmentation of the volume of interests

**FIGURE 2:**
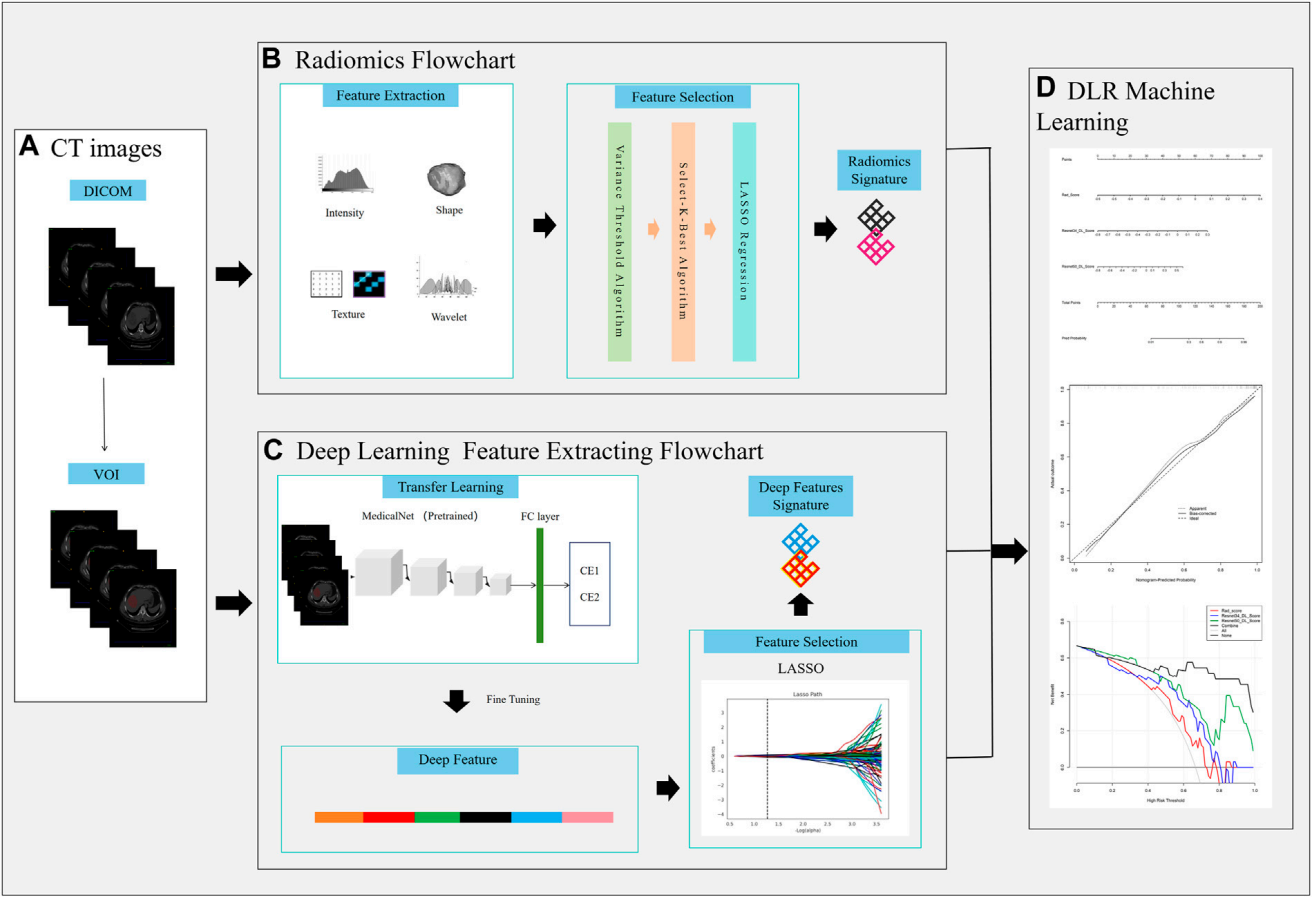
Workflow chart of the study. **(A)** VOI delineation, **(B)**, Radiomics signature construction, **(C)** Deep learning signature construction, **(D)** DLR nomogram construction.

The work flow chart of our research is shown in Figure 2. The data was uploaded to the artificial intelligence research platform of Huiying Medical Technology (Beijing) Co., Ltd. A semi-automatic method was employed to delineate the hepatic hydatid lesion on the CT image. Each slice’s associated lesion must be delineated. A physician with 3 years of expertise in chest CT image diagnosis (radiologist 1) completed all of the drawing work, when multiple lesions were encountered, we selected the largest of them for lesion delineation, and a physician with 10 years of experience in chest CT image diagnosis (radiologist 2) assessed the findings. The VOI was manually segmented based on the lesion boundary, carefully excluding areas of necrosis, cystic degeneration, hemorrhage, calcification, and edema to minimize misidentification as parasitic lesion. The data that passed the review were used for feature extraction, and the data that failed the review were discussed and redrawn by the two doctors. All data were randomly grouped, and the training and validation set had 127 and 33 cases respectively.

Figure 2 the workflow of the Deep Learning Radiomics (DLR) model construction.

### 2.4 Feature extraction and selection

Radiomics features were extracted from each Volume of Interest (VOI) across four categories. The first category included first-order statistical features, which reflected the overall information of VOI, like kurtosis, variance, energy, skewness, and uniformity. The second category was shape features, which reflected the 2D size and shape of each VOI, such as sphericity, surface area, maximum 2D diameter, etc. The gray-level run-length matrix (GLRLM), gray-level dependence matrix (GLDM), gray-level co-occurrence matrix (GLCM), neighborhood gray-level dependence matrix (NGLDM), and gray-level size zone matrix (GLSZM) were among the textural features that made up the third category in this study. The intensity and texture characteristics created by filtering and wavelet transforming the original CT images were included in the fourth category of higher-order statistical features. We retrieved 1,688 features overall from each VOI using Huiying Medical Technology’s Radcloud platform.

We chose the MedicalNet transfer project as the transfer learning model in this study (Chen et al., 2019) and adjusted the output the ResNet model in the MedicalNet project to facilitate the output of the deep features. The specific adjustment was to add linear classification, convolutional and pooling layer before the original the output the ResNet model. The deep features were extracted from the last convolutional layer of the model.

The MedicalNet project has amassed a medical image dataset from various projects, thereby creating a relatively extensive dataset. Leveraging this dataset, a series of 3D-ResNet pre-trained models have been provided. 3D-ResNet mainly consists of four residual blocks with shortcut connections, simulating the computational neurons and links in the brain. In this study, we used 3D-ResNet-34 and 3D-ResNet-50 as pre-trained models. Compared with traditional 2D models, 3D models can learn high-level features of three-dimensional space on CT images. At the same time, due to the small amount of data in this project, in order to avoid overfitting, 3D-ResNet-34 and 3D-ResNet-50 with fewer parameters are more suitable than 3D-ResNet- 101 and 3D-ResNet152. During the fine-tuning, we freeze the parameters of the vast majority of network layers and only train the last residual module and the classification layer. In addition, we employed the Adam optimizer with a learning rate of 0.0001 and the cross-entropy loss function. We conducted 20 epochs of training and selected the epoch with the lowest validation set loss as our final model. Our work was implemented using the Pytorch 1.8.0, and the models were trained on a NVIDIA 3060Ti GPU.

In this study, optimal features were selected from the training set. Before the feature selection procedure, all radiomics and deep characters were standardized using the Standard Scaler function, which normalizes the features by subtracting the mean and dividing by the standard deviation. Despite this, there remained a large number of radiomics and deep features. To get rid of duplicate features, we applied the variance threshold algorithm (with a variance threshold of 0.8) and the Select-K-Best technique. *P*-values less than 0.05 were utilized by the Select-K-Best method to identify the best characteristics. To cut down on a lot of duplication and irrelevant information, the least absolute shrinkage and selection operator (LASSO) regression approach was employed. Using least average mean square error (MSE) and 10-fold cross-validation in the training set with a maximum iteration of 2000, the ideal α—the coefficient of regularization in the LASSO algorithm—was chosen. Then, in the LASSO model produced by the whole training set with the ideal α, radiomics and deep features with non-zero coefficients were chosen.

$$y=\left(\frac{1}{2*N}\right)*{\left\|y-Xw\right\|}^{2}+\alpha *\left\|w\right\|$$

Where X is the features, N is the sample number, y is the sample vector marker, and α ∗ w is the regularization term.

### 2.5 Rad_Score and Deep_Score building

The Rad_Score and Deep Score for each patient were calculated using a specific formula. These scores represent comprehensive encapsulations of the radiomics features and deep features, respectively, and were utilized for subsequent model construction. The formula for calculating Rad_Score is:

Rad_Score=Intercept+radiomics_features_ 1×coefficient_ 1+

…radiomics_features_i×coefficient_i+…radiomics_features_n×coefficient_n

where n is the total number of optimal radiomics features, Intercept is the LASSO algorithm intercept, coefficient_i is the LASSO algorithm coefficient of the i-th feature, and radiomics_feature_i is the i-th optimal feature.

The formula for calculating Deep_Score is:

Deep_Score = Intercept + deep_features_ 1 × coefficient_ 1 + deep_features_i × coefficient_i + deep_features_n × coefficient_n here n is the total number of optimal deep features, Intercept is the LASSO algorithm intercept, coefficient_i is the LASSO algorithm coefficient of the *i*th feature, and deep_feature_i is the *i*th optimal deep feature.

### 2.6 Construction of the biological activity grading model

In this study, we used features from three different categories to build our model: i) radiomics features; ii) 3D-ResNet-34 deep features; iii) 3D-ResNet-50 deep features. We used the optimal radiomics features and two deep learning features based on logistic regression algorithm to construct seven models for diagnosing the biological activity grading, namely, RadScore model, 3D-ResNet-34 model, 3D-ResNet-50 model, Rad_Score and 3D-ResNet-34 model, RadScore and 3D-ResNet-50 model, 3D-ResNet-34 and3D-ResNet-50 model and RadScore and 3D-ResNet-34 and3D-ResNet-50 model (Combined model).

Establishing a nomogram using Rad_Score and two Deep_Scores (Resnet34_DL_Score, Resnet50_DL_Score) holds significant value. To assess the degree of agreement between the nomogra’s predictions and the actual outcomes in the training and validation sets, a calibration curve was built. In order to evaluate the net benefits in the training and validation sets, decision curve analysis (DCA) was utilized.

### 2.7 Statistical analysis

Clinical information was statistically analyzed using R version 4.1.0. Data modeling and analysis were performed using Python language (version 3.6.5) and the corresponding open-source libraries Pyradiomics (version 3.0. 1) and scikit-learn (version 0.19.2). Precision, accuracy, specificity, sensitivity, and the area under the receiver operating characteristic (ROC) curve were used to evaluate the model’s diagnostic performance.

## 3 Results

### 3.1 Baseline characters

We collected 12 clinical features from the patients, such as sex, symptoms (The clinical symptoms of hepatic echinococcosis patients encompass liver-related manifestations such as right upper abdominal pain or discomfort, palpable masses or lumps in the liver region, and abnormal liver function including jaundice and elevated transaminases. Additionally, systemic symptoms can include fever, fatigue, weight loss, reduced appetite, nausea, and vomiting), comorbidities, age, diameter of the lesion, ALT, glutamyl transpeptidase, albumin, prothrombin time, RBC, HB, neutrophil count and CRP level. The results of the statistical analysis indicated that there was no significant difference in these characteristics between the two groups. The statistical outcomes of these twelve characteristics across different metastatic groups are presented in Table 1.

**TABLE 1:** Characteristics of the CE1 patients and CE2 patients.

Characteristic	Active	Non-active	*P-value*
Number	109	51	
Sex (%)			0.332
Male	43 (39.4)	25 (49.0)	
Female	66 (60.6)	26 (51.0)	
Symptoms (%)			0.75
0	58 (53.2)	26 (51.0)	
1	51 (46.8)	25 (49.0)	
Comorbidities (%)			0.718
0	93 (85.3)	42 (82.4)	
1	14 (12.8)	7 (13.7)	
2	2 (1.8)	2 (3.9)	
Age [median (IQR)]	33.00 [25.00, 45.00]	40.00 [24.00, 58.00]	0.133
Lesion diameter [median (IQR)]	10.00 [8.00, 12.00]	10.00 [8.00, 12.00]	0.945
ALT [median (IQR)]	16.70 [13.40, 25.00]	15.70 [13.00, 24.90]	0.355
Glutamyl transpeptidase [median (IQR)]	19.00 [13.00, 29.00]	19.00 [12.00, 35.00]	0.969
Albumin [median (IQR)]	40.80 [37.60, 43.90]	40.30 [37.40, 43.00]	0.371
Prothrombin time [median (IQR)]	11.90 [11.10, 12.90]	11.60 [11.10, 13.80]	0.696
RBC [median (IQR)]	4.63 [4.26, 5.07]	4.77 [4.44, 5.01]	0.284
HB [median (IQR)]	130.00 [118.00, 145.00]	132.00 [122.00, 144.50]	0.686
Reutrophil count [median (IQR)]	0.82 [0.49, 3.71]	0.69 [0.45, 2.90]	0.285
CRP [median (IQR)]			
Number	0.48 [0.00, 1.05]	0.50 [0.00, 1.19]	0.83

### 3.2 Selected results of radiomics and deep features

**FIGURE 3:**
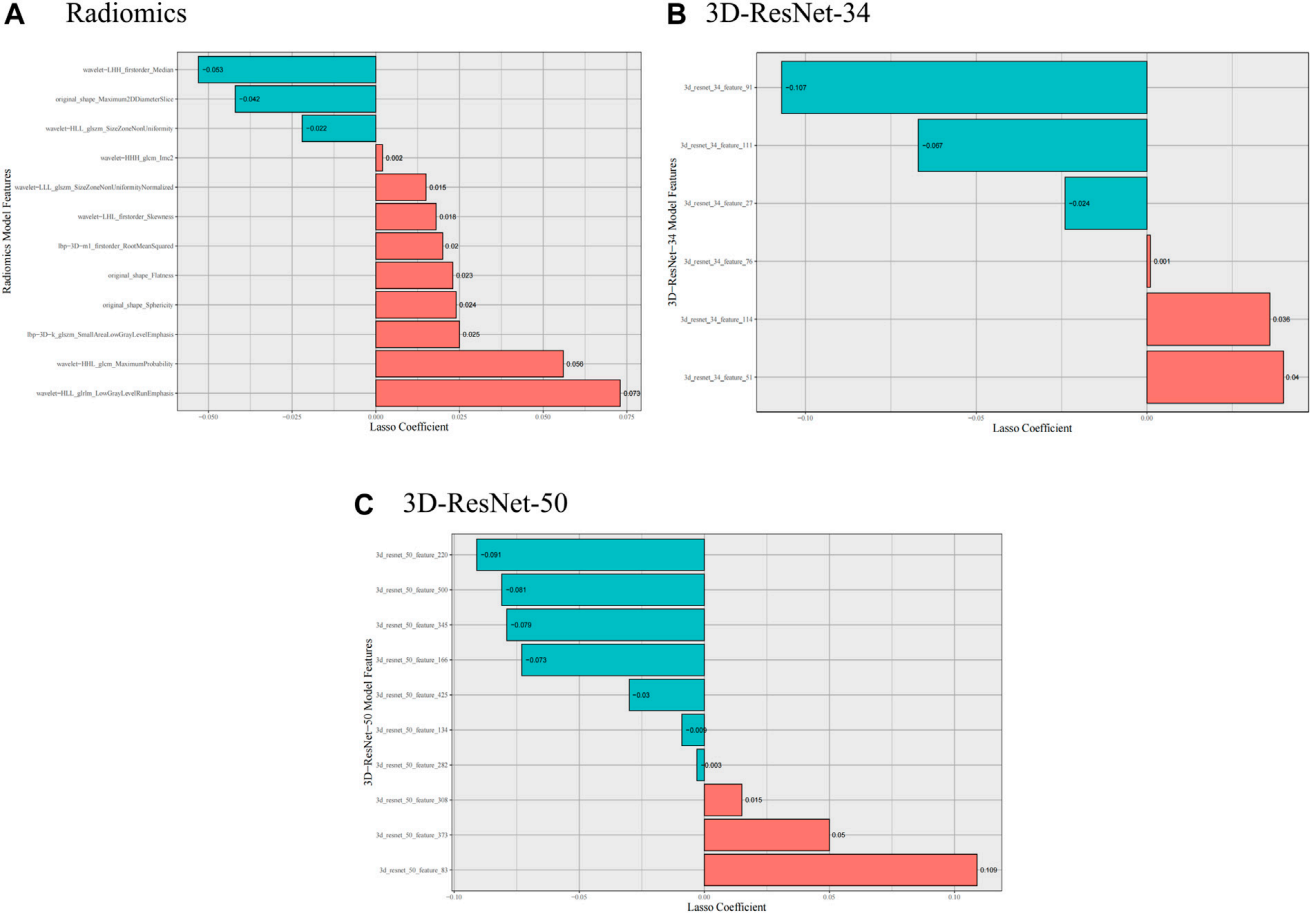
Selected radiomics and deep learning features. **(A)** Selected radiomics features, **(B)** Selected 3D-ResNet-34 features, **(C)** Selected 3D-ResNet-50 features.

From the VOI of each patient, we obtained 1,688 radiomic features 3D-ResNet-34 and 3D-ResNet-50 deep learning features. We applied feature selection algorithms to identify the optimal features. Finally, we selected 12 radiomics features, 6 3D-ResNet-34 deep learning features and 10 3D-ResNet-50 deep learning features as the optimal features set (Figure 3).

### 3.3 Evaluation of the predication models

**FIGURE 4:**
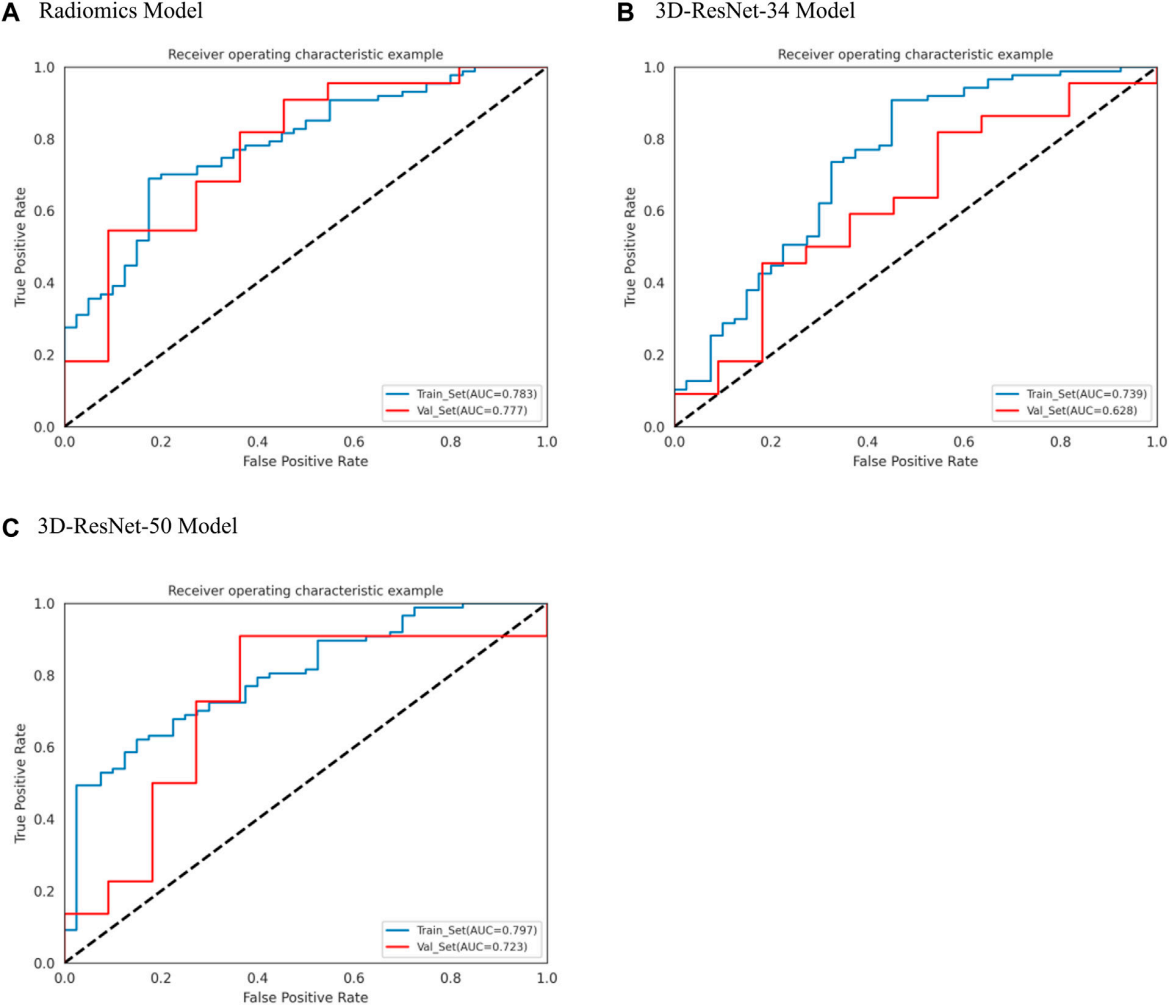
ROC curves of radiomics, 3D-ResNet 34 and 3D-ResNet 50. **(A)** ROC curve of radiomics model, **(B)** ROC curve of 3DResNet 34 model, **(C)** ROC curve of 3DResNet 50 model.

**FIGURE 5:**
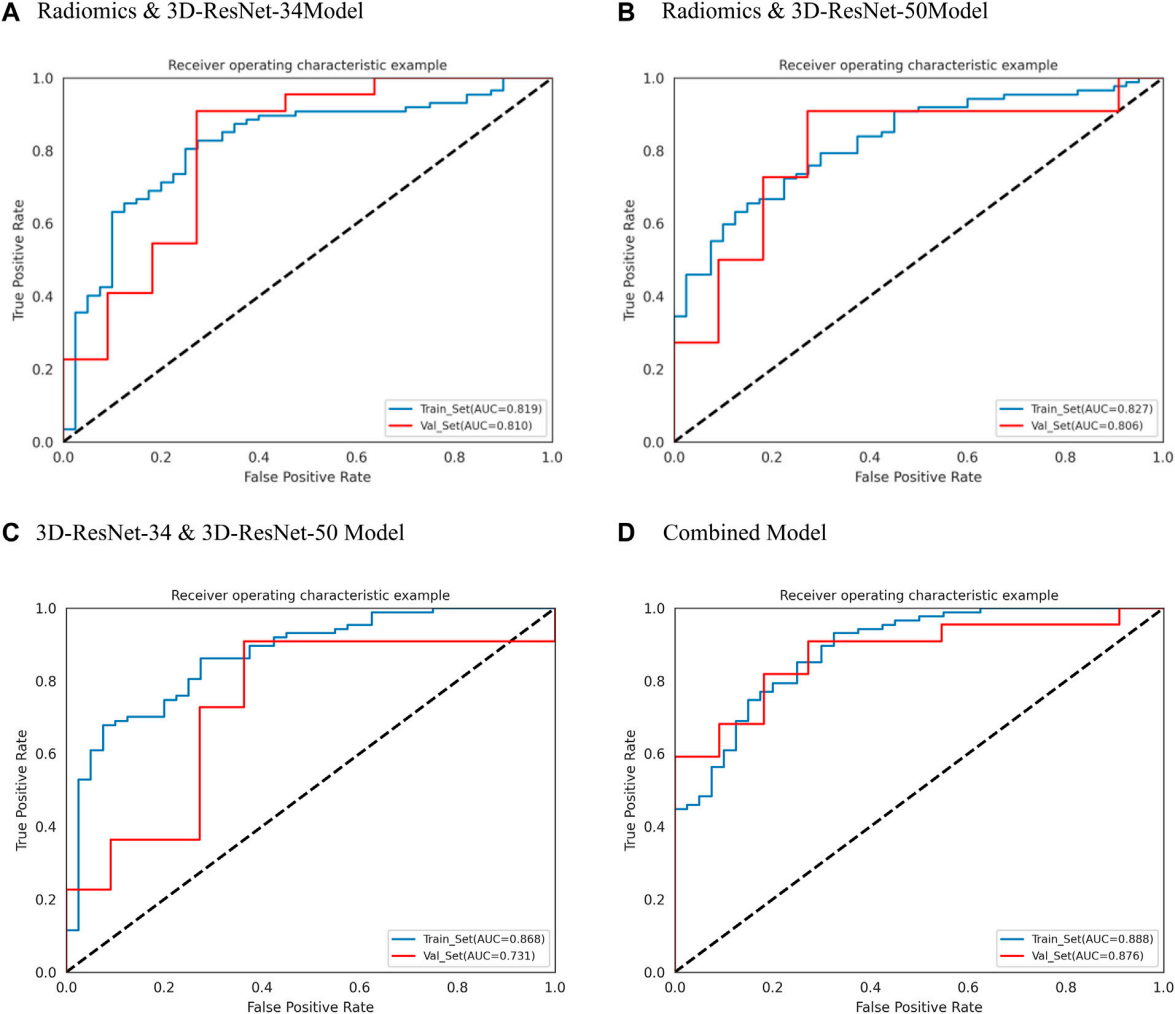
ROC curves of radiomics and 3D-ResNet 34, Radiomics and 3D-ResNet 485 50, 3D-ResNet 34 and 3D-ResNet 50 and combined model. **(A)** ROC curve of radiomics and 3DResNet 34 model, **(B)** ROC curve of Radiomics and 3DResNet 50 model, **(C)** ROC curve of 3DResNet 34 and 3DResNet 50 model, **(D)** ROC curve of combined model.

We used the optimal radiomics features and two deep learning model features based on logistic regression algorithm to construct seven models. Table 2 shows the specific evaluation metrics of all models, and Figures 4, 5 show the ROC curves of all 245 models.

**TABLE 2:** Evaluation table of all models of the biological activity grading.

Model	Data set	AUC	Accuracy	Precision	Sensitivity	Specificity
Radiomics	Training set	0.783 (0.709–0.847)	0.709	0.847	0.701	0.725
	Validation set	0.777 (0.630–0.921)	0.667	0.824	0.636	0.727
3D-ResNet-34	Training set	0.739 (0.653–0.820)	0.669	0.817	0.667	0.675
	Validation set	0.628 (0.434–0.790)	0.628	0.727	0.727	0.455
3D-ResNet-50	Training set	0.797 (0.728–0.861)	0.701	0.866	0.667	0.775
	Validation set	0.723 (0.514–0.877)	0.606	0.846	0.500	0.818
Radiomics and 3D-ResNet-34	Training set	0.819 (0.741–0.883)	0.795	0.851	0.851	0.675
	Validation set	0.810 (0.655–0.946)	0.848	0.870	0.909	0.727
Radiomics and 3D-ResNet-50	Training set	0.827 (0.763–0.889)	0.748	0.824	0.805	0.625
	Validation set	0.806 (0.654–0.938)	0.818	0.864	0.864	0.727
3D-ResNet-34 and 3D-ResNet-50	Training set	0.868 (0.808–0.918)	0.764	0.880	0.759	0.775
	Validation set	0.731 (0.533–0.880)	0.670	0.800	0.727	0.636
Combined model	Training set	0.888 (0.837–0.936)	0.780	0.893	0.770	0.800
	Validation set	0.876 (0.761–0.964)	0.818	0.900	0.818	0.818

Among the models with single input as predictors for diagnosing the biological activity grading, the radiomics model, 3D-ResNet-34 model and 3D-ResNet-50 model had AUC values of 0.777 (95% CI: 0.630–0.921), 0.628 (95% CI: 0.434–0.790) and 0.723 (95% CI: 0.514–0.877) in the validation set; accuracy of 0.667, 0.628, 0.606; precision of 0.824, 0.727, 0.846; sensitivity of 0.636, 0.727, 0.500; specificity of 0.727, 0.455, 0.818, respectively. Among the three models, the radiomics model demonstrated the best diagnostic performance, achieving a balanced identification of CE1 and CE2.

In the case of the diagnostic models that utilized two inputs to predict the biological activity grading, the validation set AUC values were as follows: 0.810 (95% CI: 0.655–0.946) for the RadScore and3D-ResNet-34 model, 0.806 (95% CI: 0.654–0.938) for the RadScore and3D-ResNet-50 model, and 0.731 (95% CI: 0.533–0.880) for the 3DResNet-34 and 3D-ResNet-50 model. Additionally, the accuracy, precision, and specificity values were 0.848, 0.818, and 0.670, respectively; sensitivity, 0.864, and 0.727, respectively; and specificity, 0.727, 0.727, and 0.636, respectively. The combined models, which used two inputs as predictors, demonstrated a significant improvement in diagnostic performance compared to the models that used a single input as a predictor. Notably, the combined model that incorporated both radiomics and deep learning features exhibited the best performance.

**FIGURE 6:**
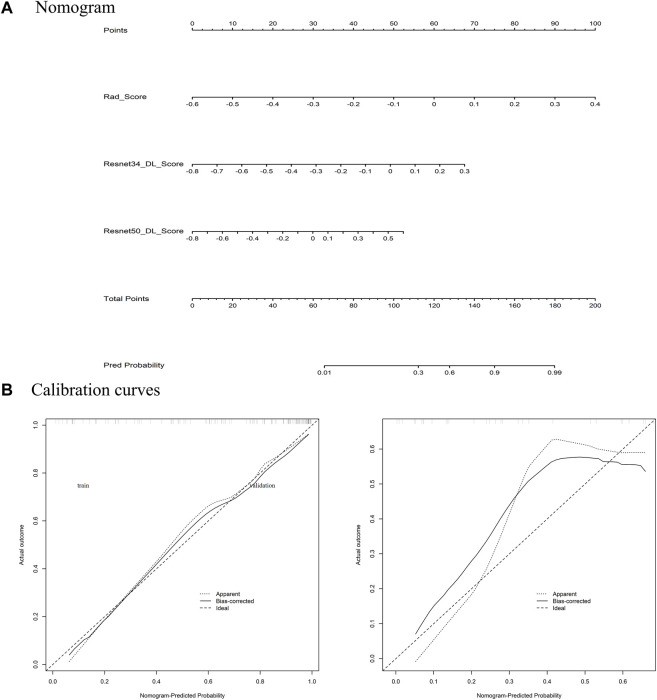
**(A)** Constructed Nomogram; **(B)** calibration curve.

**FIGURE 7:**
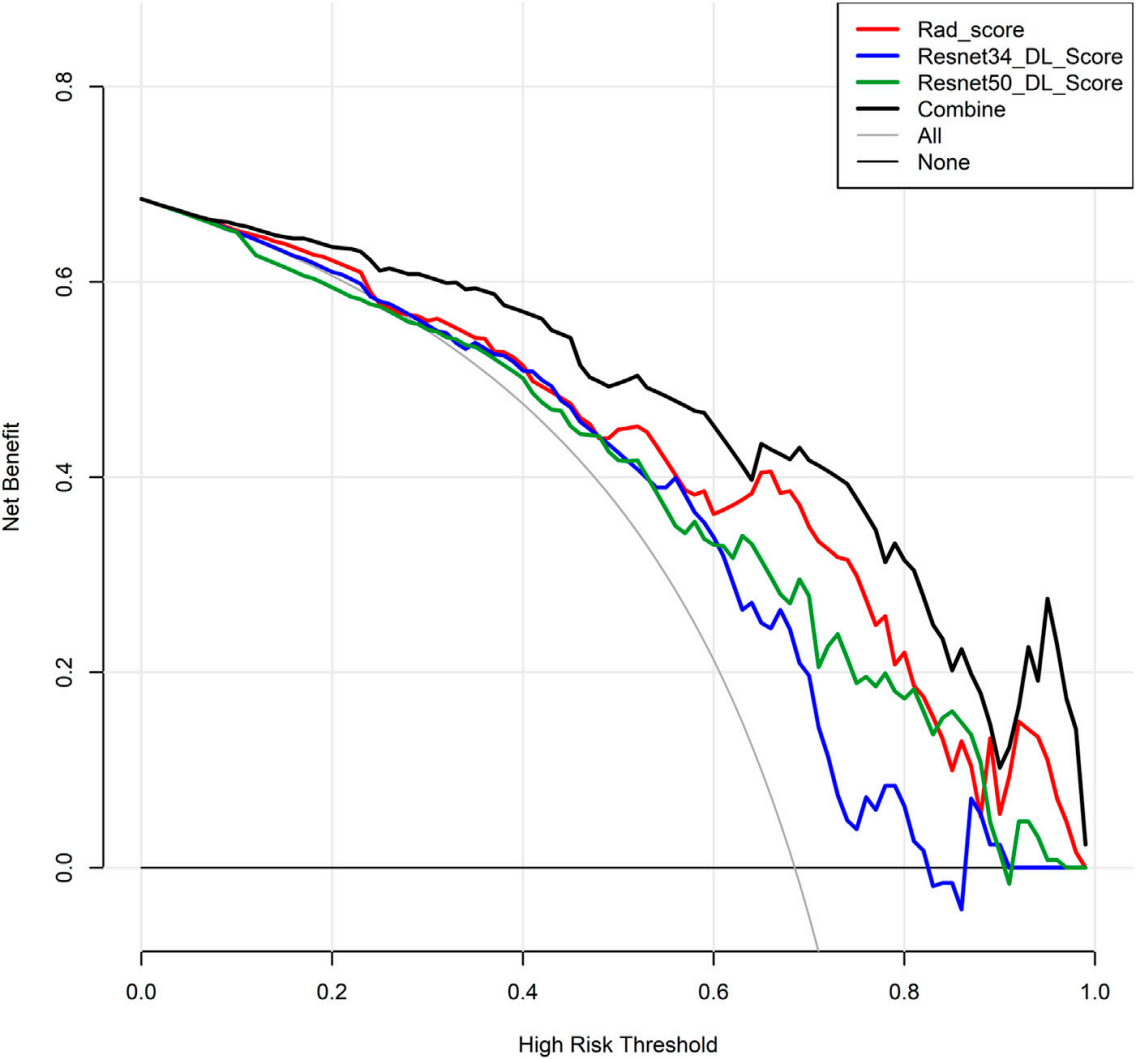
Decision curve.

Among the models with three factors as predictors, the model achieved an AUC value of 0.876 (0.761–0.964), an accuracy of 0.818, a precision of 0.900, a sensitivity of 0.818, and a specificity of 0.818 in the validation set. The combined model with three inputs demonstrated the best diagnostic performance among all models, with both sensitivity and specificity exceeding 80%, and precision reaching 90%. By merging the Rad_Score and Deep_Scores in the training set, a nomogram based on the combined model was created (Figure 6A). The calibration curve showed that in both the training and validation sets, the actual biological activity grading result was compatible with the anticipated probability of the nomogram (Figure 6B). According to DCA (Figure 7), when the threshold was more than 0. One in the training set and greater than 0.45 in the validation set, the nomogram model outperformed the other models in terms of net benefit.

## 4 Discussion

In this study, we retrospectively constructed a non-invasive prediction model for biological activity grading (active and non-active) based on artificial intelligence methods using CT images. By extracting high-throughput radiomics features and two 3D-RestNet deep learning features from the hepatic hydatid lesion area, we performed multifactor logistic regression modeling and a nomogram to diagnose the grading of hepatic hydatid activity. The final radiomics feature combined with deep learning feature diagnosis model (the combined model) achieved a good diagnostic performance, with an AUC of 0.876 (0.761–0.964) in the validation set and 0.888 (95% CI: 0.837–0.936) in the training set.

The parasitic infection known as liver hydatidosis, or hepatic hydatid cyst, has been passed on by the tapeworm larvae Echinococcus granulosus. This illness is widespread in poor nations all over the world and poses an existential threat to public health in areas where it is prevalent. Humans get the parasite’s larvae most commonly via consuming contaminated food, drink, or environment that has been exposed to the parasite’s eggs (Agudelo Higuita et al., 2016; Li et al., 2020). Echinococcus granulosus larvae mostly inhabit dogs, with sheep, goats, and cattle acting as intermediary hosts. By consuming the parasite’s eggs, humans unintentionally become intermediate hosts. The parasite’s larvae then travel through the portal vein to the liver, where they develop into hydatid cysts (Kern et al., 2017).

Accurate distinguishing between active and non-active hepatic echinococcosis is crucial for appropriate management and treatment decisions (International classification of ultrasound images, 2003). The management approach for active and non-active hepatic echinococcosis exhibits notable disparities. Active disease typically necessitates a combination of medical therapy and surgical intervention to mitigate disease progression and associated complications. Conversely, non-active manifestations may warrant vigilant monitoring or less invasive therapeutic modalities (Apaer et al., 2021).

Active hepatic echinococcosis imposes a heightened susceptibility to adverse outcomes, including cyst rupture, secondary infection, and systemic dissemination of the parasitic agent. Consequently, patients with active disease often face a graver prognosis, particularly if complications have ensued. Furthermore, active disease carries an augmented risk of recurrence post-treatment compared to its non-active counterpart (Bhutani and Kajal, 2012; Joliat et al., 2015).

Precise discernment of disease activity enables healthcare providers to customize treatment strategies aimed at mitigating recurrence risk and optimizing patient outcomes. Moreover, accurate identification of parasite activity facilitates effective patient-provider communication, facilitating the development of comprehensive treatment and follow-up plans tailored to individual patient needs (Balli et al., 2019).

In recent years, artificial intelligence algorithms have rapidly developed and achieved many successes in medical image data mining, such as the application of radiomics in ^26^such as clinical auxiliary diagnosis, surgical path planning, lesion segmentation and measurement, etc. In previous studies, Ren (Ren et al., 2021) established a radiomics model to predict the biological activity of hepatic hydatid based on MRI images, and the optimal 322 diagnostic model had a prediction performance of AUC = 0.830 ± 0.053 in the validation set.

The features extracted in radiomics mainly include shape features, texture features, intensity features and filtering features, with thousands of quantitative features. However, the quantitative features of radiomics have some limitations, as their design completely depends on human prior knowledge, which makes it very likely that radiomics features do not contain important features that humans have not discovered. Therefore, the features of deep learning with self-learning ability are particularly necessary. Deep learning networks can mine hidden features that reflect the prediction events from a higher level, and can extract quantifiable higher-level hidden features that humans do not know. The DLR method that combines deep learning features with radiomics features will enrich the feature set of the diagnostic model and ensure the reliability of the model from the dimension of data input (Shao et al., 2021; Zhou et al., 2021).

Deep learning models trained from scratch are prone to overfitting when applied to a specific clinical problem with limited data. In the medical field, using pre-trained convolutional neural networks (CNN) as feature extractors has been considered an effective way to overcome this difficulty. Transfer learning can transfer the image feature extraction method from the pre-trained model to a new model, with advantages such as better generalization and ease of replication (Shin et al., 2016; Lopes and Valiati, 2017; Zhu et al., 2019; Hu et al., 2021). There are few open-source medical image pre-trained models available, which leads to the previous DLR studies using 2D pre-trained networks. Such as Yihuai Hu who extracted six pre-trained CNN deep learning features from CT images and used support vector machine as a classifier to study esophageal squamous cell carcinoma pathological complete response to neoadjuvant radiochemotherapy, with a prediction performance of AUC value of 0.805 in the test set. 2D pre-trained models lack the exploration of 3D features on medical images compared to 3D pre-trained models, and cannot describe the continuity of lesion features between medical image slices. In this study, we used 3D pre-trained deep learning networks to extract 3D deep learning features using transfer learning methods, and combined with traditional radiomics features to construct a model for distinguishing hepatic hydatid activity grading, achieving a satisfactory result.

The model proposed in this study has practical implications in clinical practice, aiding radiologists and treating physicians in making decisions regarding patient management. By assisting in determining the need for discharge with medication, follow-up care, or further diagnostic assessments and treatment, the model can enhance diagnostic support and efficiency in abdominal CT analyses. This has the potential to optimize patient care by reducing unnecessary examinations and facilitating timely and effective treatment interventions.

### 4.1 Limitations

However, this study still has some limitations. First, the small number of patients included in the study due to the difficulty of collecting cases, in addition data imbalance, which affects model performances, in our further studies, we will incorporate more cases to tackle the problems. Second, this study did not include data from external centers to evaluate the DLR model with external validation, and we plan to collect data from external centers to improve this study in the future. Third, in this study, model explanation techniques were not utilized. However, in our upcoming research, we plan to employ these techniques to address the black-box nature of deep learning models. Fourth, due to the relatively small sample size in this study, issues such as overfitting or underfitting are inevitable. In our future studies, we plan to increase the sample size to mitigate these challenges.

In summary, we established a non-invasive prediction model for hepatic hydatid activity grading based on the DLR methods, and achieved good predictive performance. Our prediction model will help identify the grading of hepatic hydatid activity and guide personalized treatment plans.

## 5 Conclusion

We achieved good performance in predicting the activity of hepatic echinococcosis patients using the classifier based on the DLR model derived from CT images. Our model is a potential clinical tool that can assist in guiding personalized treatment plans for the patients.

## Data Availability

The original contributions presented in the study are included in the article/Supplementary Material, further inquiries can be directed to the corresponding authors.
